# Antioxidant Extracts from Greek and Spanish Olive Leaves: Antimicrobial, Anticancer and Antiangiogenic Effects

**DOI:** 10.3390/antiox13070774

**Published:** 2024-06-27

**Authors:** Ioana Zinuca Magyari-Pavel, Elena-Alina Moacă, Ștefana Avram, Zorița Diaconeasa, Daniela Haidu, Mariana Nela Ștefănuț, Arpad Mihai Rostas, Delia Muntean, Larisa Bora, Bianca Badescu, Cristian Iuhas, Cristina Adriana Dehelean, Corina Danciu

**Affiliations:** 1 Department of Pharmacognosy, “Victor Babeș” University of Medicine and Pharmacy Timișoara, Eftimie Murgu Square, No. 2, 300041 Timisoara, Romania; ioanaz.pavel@umft.ro (I.Z.M.-P.); larisa.bora@umft.ro (L.B.); corina.danciu@umft.ro (C.D.); 2 Department of Toxicology, Drug Industry, Management and Legislation, Faculty of Pharmacy, “Victor Babeș” University of Medicine and Pharmacy Timișoara, Eftimie Murgu Square, No. 2, 300041 Timisoara, Romania; alina.moaca@umft.ro (E.-A.M.); cadehelean@umft.ro (C.A.D.); 3 Research Centre for Pharmaco-Toxicological Evaluation, “Victor Babeș” University of Medicine and Pharmacy Timișoara, Eftimie Murgu Square, No. 2, 300041 Timisoara, Romania; 4 Department of Food Science and Technology, Faculty of Food Science and Technology, University of Agricultural Science and Veterinary Medicine, Calea Manastur, 3-5, 400372 Cluj-Napoca, Romania; zorita.sconta@usamvcluj.ro; 5 Romanian Academy “Coriolan Dragulescu” Institute of Chemistry, Bv. M. Viteazu, No. 24, 300223 Timisoara, Romania; danielahaidu1@gmail.com; 6 Department of Chemical and Electrochemical Syntheses, Laboratory of Electrochemical and Chemical Technologies, National Institute of Research and Development for Electrochemistry and Condensed Matter, Dr. A. P. Podeanu 144, 300569 Timişoara, Romania; mariana.stefanut@gmail.com; 7 National Institute for Research and Development of Isotopic and Molecular Technologies-INCDTIM, 67-103 Donat, 400293 Cluj-Napoca, Romania; arpad.rostas@itim-cj.ro; 8 Department of Microbiology, “Victor Babeș” University of Medicine and Pharmacy Timișoara, Eftimie Murgu Square, No. 2, 300041 Timișoara, Romania; muntean.delia@umft.ro; 9 Doctoral School, “Victor Babeș” University of Medicine and Pharmacy Timișoara, Eftimie Murgu Square, No. 2, 300041 Timișoara, Romania; bianca.badescu@medicis.ro; 10 Department of Obstetrics and Gynecology, Faculty of Medicine, Iuliu Hatieganu University of Medicine and Pharmacy, Victor Babes Street No. 8, 400012 Cluj-Napoca, Romania; iuhascristianioan@yahoo.co.uk

**Keywords:** olive leaves extracts, phenolic compounds, pentacyclic triterpenes, inorganic elements, antioxidant, antimicrobial, melanoma cells, keratinocytes, chorioallantoic membrane, angiogenesis

## Abstract

*Olea europaea* L. is the most valuable species of the *Olea* type, and its products offer a wide range of therapeutical uses. The olive tree has been extensively studied for its nourishing qualities, and the “Mediterranean diet”, which includes virgin olive oil as a key dietary component, is strongly associated with a reduced risk of cardiovascular disease and various malignancies. Olive leaves, a by-product in the olive harvesting process, are valued as a resource for developing novel phytomedicines. For this purpose, two ethanolic extracts obtained from *Olivae folium* from Spain (OFS) and Greece (OFG) were investigated. Our findings contribute to a wider characterization of olive leaves. Both extracts displayed important amounts of phenolic compounds and pentacyclic triterpenes, OFG having higher concentrations of both polyphenols, such as oleuropein and lutein, as well as triterpenes, such as oleanolic acid and maslinic acid. The antioxidant capacity is similar for the two extracts, albeit slightly higher for OFG, possibly due to metal polyphenol complexes with antioxidant activity. The extracts elicited an antimicrobial effect at higher doses, especially against Gram-positive bacteria, such as *Streptococcus pyogenes*. The extract with lower inorganic content and higher content of polyphenols and triterpenic acids induced a strong anti-radical capacity, a selective cytotoxic effect, as well as antimigratory potential on A375 melanoma cells and antiangiogenic potential on the CAM. No irritability and a good tolerability were noted after evaluating the extracts on the in vivo Hen’s Egg Test−Chorioallantoic Membrane (HET-CAM). Therefore, the present data are suggestive for the possible use of the two types of olive leaf products as high-antioxidant extracts, potentially impacting the healthcare system through their use as antimicrobial agents and as anticancer and anti-invasion treatments for melanoma.

## 1. Introduction

Appraisal of the latest scientific trends has led to the conclusion that phytochemicals produced by medicinal plants serve as pivotal resources for developing new medicines, especially chemotherapeutics [1]. Notable examples of lead compounds are plant-based medicines already placed on the pharmaceutical market as pain relievers, anti-malarial drugs, and anti-hypertensive or antimicrobial agents [2,3]. Phytochemicals extracted from various plants (*Catharanthus roseus* L. G. Don, *Podophyllum peltatum* L., *Taxus brevifolia* Nutt., *Taxus baccata* L., etc.) have found their way into the oncology area, where they have been licensed for the treatment of different malignancies, such as leukemia, Hodgkin’s lymphoma, lung cancer, and breast cancer [4,5]. Thus, several novel cytotoxic and chemopreventive agents are being discovered within the plant kingdom annually.

In this current scenario, medicinal plants, especially those with anti-melanoma activity, have sparked the interest of researchers, as melanoma is one of the most aggressive forms of skin cancer. The distinctive feature of this type of cancer is represented by its high tendency for metastasis. Melanoma is also related to UV exposure due to DNA damage [6]. Despite the significant advances in diagnosis and treatment, this malignancy has dismal outcomes [7]. In this context, phytochemicals are being intensively studied as potentially safer and more successful targeted therapies for melanoma. Numerous human diseases, including cancer, have been linked to oxidative stress, while molecules known as antioxidants can lower oxidative stress levels, which may inhibit the growth of cancer. Among phytochemicals, secondary metabolites, such as phenolic compounds, contribute the most to the antioxidant properties of foods and herbal products. Flavonoids are one of the most representative secondary plant metabolites subjected to extensive research in the field of oncology. Flavonoids, such as kaempferol, quercetin, apigenin, luteolin, butein, naringenin, isoliquiritigenin, genistein, and daidzein, have been shown to exhibit in vitro antiproliferative and proapoptotic activities against the B164A5 melanoma cell line [8]. By regulating the *β*3 integrin and the epithelial-mesenchymal transition, luteolin has been shown to reduce the invasive potential of the murine B16F10 melanoma cell line [9]. A recent study conducted by Ghitu et al. employing in vitro and in vivo evaluation of apigenin on the SK-MEL-24 human melanoma cell line has depicted anti-migratory, cytotoxic, and antiproliferative potential, thus limiting melanoma cell development [10]. 

Extensive research into the anticancer properties of various medicinal plants has concluded that *Olea europaea* L. products, including extracts and specific phytochemicals, offer a wide range of therapeutical uses. The genus *Olea* encompasses 30 species, although *Olea europaea* L. is the most valuable member of the genus [11,12]. The olive tree is economically important in the Mediterranean region, serving as a significant supply of olive oil. It is native to the eastern Mediterranean basin’s coastal regions and the adjacent coastal regions of southeastern Europe, western Asia, the Arabian Peninsula, India, Asia, and northern Africa. *Olea europaea* L. has been extensively studied for its nourishing qualities. Further to the nutritional value, olive leaves are valued as a resource for developing novel phytomedicines. Olive tree leaves contain polyphenols comparable to those found in olive oil or fruit but in a significantly higher proportion. As a result, olive leaf extract (OLE) may have much more potential for enhancing health outcomes than virgin oil [13]. Olive leaves comprise five categories of phenolic compounds: oleuropeosides (oleuropein and verbascoside); flavones (luteolin-7-glucoside, apigenin-7-glucoside, diosmetin-7-glucoside, luteolin, and diosmetin); flavonols (rutin); flavan-3-ols (catechin); and substituted phenols (tyrosol, hydroxytyrosol, vanillin, vanillic acid, and caffeic acid). Oleuropein is the most common phytocompound in olive leaves, followed by hydroxytyrosol, luteolin, apigenin flavone-7-glucosides, and verbascoside [14]. The total phenol content of OLEs ranges from 73.05 to 144.19 mg gallic acid equivalents/g (GAE/g), whereas the total flavonoid content ranges from 56.75 to 125.64 mg CE/g [15]. Notably, olive leaves’ polyphenol content varies depending on several variables, including cultivar, climatic conditions, and crop cycle stage [16]. Due to the high content of polyphenols, olive leaves provide various health benefits, including antimicrobial activity and antioxidant capacity, which can be associated with remediation pathways in various pathological conditions [17]. Most of the studies that evaluated the polyphenolic composition described olive leaf’s antioxidant activity, and correlations were established. The same types of extracts were evaluated for antimicrobial effects and were found active on strains such as *Bacillus cereus*, *Salmonella typhimurium*, and *Staphylococcus aureus*, among others [18,19]. Oleuropein, one of the most abundant compounds in olive leaves, was indicated as a potent agent against oxidative stress, similar to the effect exerted by ascorbic acid (vitamin C) and α-tocopherol (vitamin E) [20]. Hence, olive leaves are a valuable by-product resulting during olive harvests that can serve as a natural alternative to preservatives, gaining great interest from the pharmaceutical, cosmetic, nutraceutical, and food industries.

Apart from their preservative benefits, the antioxidant power in particular can be correlated with the latest advances in research, which demonstrated that OLE possesses a broad range of in vitro and in vivo activities, including radioprotective effects, antiproliferative and cytotoxic activity against several cancer cells, anti-HIV virus effects, and gastroprotective activity [21]. Many studies have looked into OLE’s anticancer properties, and the results are promising. Fares et al. revealed that OLE 4 µg/mL reduced cellular growth of human lymphoblastic leukemia and leukemic myelogenous cell lines [22,23]. Methanolic extract from olive leaves was shown to substantially decrease the viability of malignant cells when tested on the human breast cancer cell line MCF-7 (IC_50_ = 135 μg/mL) [24]. For instance, oral administration of 150 and 225 mg/kg/day of OLE in a breast cancer mouse model has decreased mammary cancer weight and volume [25]. Mijatovic et al. showed that methanolic extract from olive leaves exhibited strong anti-melanoma potential both in vitro and in vivo. The OLE polyphenols are deemed responsible for the anticancer properties. The phytochemical analysis of the extract revealed the following major constituents: oleuropein (19.8%), flavonoids (0.29%), including luteolin-7-O-glucoside (0.04%), apigenin-7-O-glucoside (0.07%), and quercetin (0.04%), as well as tannins (0.52%). After 48 h of treatment with 1.25 mg/mL OLE, the proliferation of murine melanoma B16 cells was strongly inhibited. Moreover, OLE has reduced tumor volume in melanoma-induced C57BL/6 mice at 40 mg/kg. The study’s authors have reported that OLE effectively induces cell death, mostly via the disintegration of cell membrane integrity and late caspase-independent fragmentation of genetic material [26]. Undoubtedly, previous research studies have demonstrated that OLE is a highly effective anti-melanoma agent.

Moreover, the modulation of the angiogenic effect was also investigated in regard to OLE as an approach for pathologies with decreased angiogenesis capacity, such as wounds, and for excessive pathological angiogenesis in cancer and metastasis, among others. Ethanolic extracts from olive leaves harvested in Spain caused a pro-angiogenic reaction in low concentrations, below 50 μg/mL. In contrast, higher concentrations, especially above 140 μg/mL, induced a decrease in angiogenic capacity, promoting differentiation of endothelial cells but a low proliferative potential after 48 and 72 h post-inoculation in vitro [27]. Other in vitro and in vivo studies showed that high concentrations of OLE from Iran alone [25] or in combination with bevacizumab [28] significantly reduced the high vascular endothelial growth factor (VEGF) levels in a breast cancer model. Oleuropein, a polyphenol found in high concentrations in olive leaves, was found to contribute as a regulator of overexpressed angiogenic factors, such as VEGF-A, VEGF-C, VEGF-D, and the involved receptors, thus reducing the degree of angiogenesis and lymphangiogenesis, using in vitro and in vivo in melanoma models [29]. Tekin et al. showed that, at high concentrations, OLE can reduce vascularization in combination with chemotherapeutic agents, such as cisplatin, using the chorioallantoic membrane (CAM) assay [29]. Others indicated that extracts from olive leaves originating from Albania in concentrations above 15 μg/mL reduced tumor growth and angiogenesis in a CAM melanoma model [30]. Still, a limited number of studies have explored the angiogenesis implications of OLE using the *in ovo* choriollantoic membrane assay, a cost- and time-efficient experimental alternative to murine protocols. 

Olive leaves, as a herbal source rich in several types of active compounds, from various geographic areas, are currently being investigated in order to obtain the optimal types of extracts and investigate their biological activities, gaining mechanistic insights into which can serve as alternative treatments for pathologies confronted with a lack of efficacy, resistance or side effects, such as antimicrobial therapies or cancer [31]. Several published studies focus on the polyphenolic content and the antioxidant capacity of olive leaves as a by-product from variable olive tree cultivars from different areas around the globe, [32,33,34], and different studies explore the anticancer potential of olive leaves [35,36]; however, there are fewer publications describing the phytochemical profile in corroboration with antioxidant and antimicrobial activity and topical application intended for anti-melanoma effects. 

In this light, we intended to characterize the leaves of olive trees (*Olivae folium*, OF) from the Mediterranean climate, particularly those harvested from Seville, Spain (OFS), and Lefkada, Greece (OFG). Our study design planned to deepen the phytochemical composition of the OF ethanolic extracts, focusing on polyphenols, triterpenes, and inorganic elements in correlation with the antioxidant capacity, hence revealing potential therapeutic benefits as antimicrobial agents or anti-melanoma and angiogenesis-modulating agents with topical applications, using in vitro assays and the in ovo chorioallantoic membrane protocol.

## 2. Materials and Methods

### 2.1. Plant Materials

The olive leaves were harvested from Spain and Greece. OFS represents the leaves harvested from Seville, Spain (Picual variety), and OFG the leaves harvested from Lefkada, Greece (Koroneiki variety). OFS was assigned the voucher specimen code OFS/2018 and OFG the code OFG/2018. After drying, both plant materials were subjected to grounding, after which 5 g of dried plant material were weighed and mixed with 100 mL of aqueous ethanolic solvent (80% (*v*/*v*)). The samples were left to rest for 15 min and then subjected to ultrasound-assisted extraction (Falc LCD series) for 30 min at 40 °C, power 800 W, and frequency 40 KHz. Then, the samples were filtered and concentrated in a rotary evaporator (Heidolph Laborota 4000 efficient) at a temperature of 50 °C, 150 rpm, and 250 mbar (the pressure was gradually reduced at time intervals of 20 min to a value of 80 mbar). The dried extracts were stored at −20 °C before use. The extracts were performed in triplicate. The same procedure was applied for all extracts.

### 2.2. High-Performance Liquid Chromatography-Photo Diode Array (HPLC-PDA)/Electrospray Ionization-Mass Spectrometry (ESI-MS) Identification and Quantification of Phenolic Compounds

Phenolic compounds were identified and quantified by HPLC analysis by means of an Agilent 1200 system (Chelmsford) with a quaternary pump delivery system LC-20 AT (Prominence), a degasser DGU-20 A3 (Prominence), and a diode array SPD-M20 UV–VIS detector (DAD). MS with a mass detector single-quadrupole Agilent model 6110 (Agilent Technologies, Santa Clara, CA, USA) equipped with an ESI+ probe served for the identification of phenolic compounds.

An Eclipse XDB C18 column (4.6 × 150 mm, 5 µm), Agilent Technologies for 30 min, at 25 °C, with a 0.5 mL/min flow rate and an injection volume of 20 µL, was used for phytocompound separation. The mobile phases consisted of solvent A-distilled water and 0.1% acetic acid and solvent B-acetonitrile and 0.1% acetic acid. The gradient elution system was considered as follows: 0–2 min, isocratic with 5% (vol/vol) eluent B; 2–18 min, linear gradient from 5% to 40% (vol/vol) eluent B; 18–20 min, linear gradient from 40% to 90% (vol/vol) eluent B; 20–24 min, isocratic with 90% (vol/vol) eluent B; 24–25 min, linear gradient from 90% to 5% (vol/vol) eluent B; 25–30 min, isocratic with 5% (vol/vol) eluent B. The chromatograms were monitored at 280 nm corresponding to hydroxybenzoic acids and 340 nm for hydroxycinnamic acids and flavonoids. The identification of compounds was conducted based on their retention times, UV-VIS spectra, and comparison with commercial standards purchased from Sigma−Aldrich (St. Louis, MO, USA). For the MS analysis, measurements were performed in the positive mode with a capillary voltage of 3000 V, a temperature of 300 °C, and a collision energy of 50 eV.

Data were collected in full scan mode within 100 to 1000 *m*/*z* with an 8 L/min nitrogen flow rate. Data acquisition and interpretation were performed using Agilent ChemStation software. A standard curve of luteolin was used to quantify the phenolic compounds (concentrations between 10 and 100 µg/mL). 

### 2.3. HPLC-PDA/ESI-MS Identification and Quantification of Pentacyclic Triterpenes

For pentacyclic triterpenes chromatographic analysis, an Agilent 1200 HPLC system equipped with a quaternary pump, solvent degasser, autosampler, and UV-Vis detector with a photodiode (DAD) coupled with mass detector single quadrupole Agilent model 6110 (Agilent Technologies, CA, USA) was used.

The compounds were separated on a Kinetex XB-C18 column, dimensions 4.6 × 150 mm, with 5 μm particles (Phenomenex, Torrance, CA, USA). The mobile phase consisted of methanol/bidistilled water in a ratio of 92/8 (*v*/*v*) acidified with 0.1% formic acid, isocratic system. The separation was performed for 20 min at a temperature of 250 °C with a 0.5 mL/min flow rate. The spectral values were recorded for all peaks in the 190–300 nm range, whereas the chromatograms were recorded at λ = 210 nm. For MS, the ESI negative ionization mode was used in the following working conditions: capillary voltage: 3000, temperature: 3500 °C, nitrogen flow: 7 l/min, *m*/*z*: 120–1200, full-scan. Data acquisition and interpretation of results were carried out using the Agilent ChemStation software (Rev B.04.02 SP1, Palo Alto, CA, USA). For the quantification of the compounds, the following four standard curves were used: maslinic acid (concentrations between 0 and 500 µg/mL), betulin (concentrations between 0 and 200 µg/mL), oleanolic acid (concentrations between 0 and 1000 µg/mL), and ursolic acid (concentrations between 0 and 200 µg/mL). 

### 2.4. Inorganic Elemental Determination of OF Extracts by the Graphite Furnace-Atomic Absorption Spectrometry (GF-AAS) Method

Digestion with nitric acid (purity ≥ 65%, Sigma−Aldrich, Taufkirchen, Germany) disintegrates the vegetal matrix at 160–210 °C for 45 min in a microwave system MWS-2 (Berghof oven). Appropriate dilutions for analysis were prepared with ultrapure water (Barnstead, EASYpure RoDi^®^ apparatus). The Spectrophotometer novAA 400G (Analytik Jena, Jena, Germany) with autosampler MPE60 and Cookbook, equipped with a graphite furnace, was used to determine desired metal concentrations. A standard Merk solution was used for the calibration curve for each element, as previously registered. The analyzed data were examined with WinAAS 3.17.0 soft (Table 1).

### 2.5. Thermogravimetry-Differential Scanning Calorimetry (TG-DSC) and Fourier Transform-Infrared Spectroscopy (FT-IR) Characterization Techniques

Thermal behavior of the two olive extracts was recorded in the range 25–1000 °C in an air atmosphere at a heating rate of 10 K·min^−1^ and a flow rate of 20 mL·min^−1^ using a Netzsch STA 449C apparatus (Selb, Germany) that works with alumina crucibles.

The functional groups of both dried olive extracts were characterized by FT-IR spectroscopy using a Shimadzu Prestige-21 spectrometer (Duisburg, Germany) at room temperature with a resolution of 4 cm^−1^ in the spectral region ranging from 4000–400 cm^−1^ using KBr pellets.

### 2.6. 2,2-Diphenyl-1-Picrylhydrazyl (DPPH) Assay

The DPPH method was performed as previously described, with slight modifications [37,38] to determine the extracts’ antioxidant activity (AOA). Briefly, a volume of 0.2 mL of the OF extracts (100 and 1000 μg/mL) was mixed with 1.8 mL of 0.1 mM DPPH prepared in ethanol and further incubated for 30 min at room temperature in the dark. Ascorbic acid (100 μg/mL) was used as control. The absorbance was measured at 517 nm employing a UV-VIS spectrophotometer (PG Instruments Ltd., Lutterworth, UK). The AOA was calculated with the following formula:

$$AOA\;(\%)=\left[\frac{A_{0}-A_{s}}{A_{0}}\right]\times 100$$

where *A*_0_ is the absorbance of the blank sample and *A_s_* is the absorbance of the samples.

### 2.7. Electron Paramagnetic Resonance Spectroscopy Measurements

The Electron Paramagnetic Resonance (EPR) spectroscopy measurements were carried out with a continuous-wave Elexsys 580 EPR spectrometer (Bruker AXS GmbH, Karlsruhe, Germany) equipped with a Bruker X-SHQ 4119HS-W1 X-Band resonator. The compound’s antioxidant activity was determined with DPPH, which has a free-radical and hydrogen acceptor capability in regard to antioxidants.

### 2.8. Antimicrobial Activity

The antimicrobial activity of OFS and OFG was evaluated using both the Kirby−Bauer disk-diffusion method and broth dilution assay following the methods recommended by the Clinical Laboratory and Standard Institute (CLSI) and the European Committee on Antimicrobial Susceptibility Testing (EUCAST), also used in our previous studies [39,40,41,42,43]. For the tests, eight reference microbial strains were used, represented by *Streptococcus pyogenes* (ATCC 19615), *Staphylococcus aureus* (ATCC 25923), *Enterococcus faecalis* (ATCC 51299), *Escherichia coli* (ATCC 25922), *Salmonella enterica* sv *Typhimurium* (ATCC 14028), *Pseudomonas aeruginosa* (ATCC 27853), *Candida parapsilosis* (ATCC 22019) and *Candida albicans* (ATCC 10231) (Microbiologics, France). At first, the microbial strains were isolated on Columbia 5% sheep blood agar or Sabouraud agar (BioMerieux, Marcy-l’Étoile, France).

#### 2.8.1. Disk Diffusion Method

The Mueller−Hinton agar (BioMerieux, France), supplemented for streptococci with horse blood and β-NAD, was inoculated with microbial suspension prepared in NaCl 0.85% at 0.5 Mc Farland. Then, 10 µL from each compound at different concentrations (50 mg/mL, 25 mg/mL, 5 mg/mL, 1 mg/mL, 0.5 mg/mL) were added to a blank paper disk (BioMaxima, Lublin, Poland) and placed on top of the inoculated culture medium. The plates were incubated for 24 h at 35 °C for the bacterial strains and 28 °C for the *Candida* species. The antimicrobial activity was evaluated by measuring the diameters of the inhibition zones. For control, we used levofloxacin (LEV) and fluconazole (FCZ) disks (Bio-Rad, Marnes-la-Coquette, France), and the minimum value for the antibacterial activity of the test compounds was established at a diameter of 17 mm.

#### 2.8.2. Determination of MIC, MBC and MFC

The MICs were established by broth dilution assay using microbial suspensions of 5 × 10^5^ CFU (colony forming units)/mL, Mueller−Hinton broth (supplemented with horse blood + β-NAD for *Streptococcus pyogenes* strain), and serial two-fold dilutions of the test extracts to obtain concentrations ranging from 50 to 3.125 mg/mL. After 24 h of incubating at 28–35 °C, the MICs were determined by observing the lowest concentration of each extract that inhibited visual microbial growth. Then, the MBCs or MFCs were considered the lowest concentrations that killed 99.9% of the microorganisms and were determined by the cultivation of 1 µL suspension from test tubes with no growth on Columbia 5% sheep blood or Sabouraud agar.

### 2.9. In Vitro Evaluation

#### 2.9.1. Cell Culture

An A375 human melanoma cell line was acquired from the American Type Culture Collection (ATCC^®^ CRL-1619™). HaCaT-immortalized human keratinocytes were kindly given by the University of Debrecen, Hungary. The cells were cultured in high glucose Dulbecco’s Modified Eagle’s Medium (DMEM 4.5 g/l glucose; Sigma−Aldrich, Taufkirchen, Germany) and supplemented with a 1% penicillin/streptomycin mixture (Pen/Strep, 10.000 IU/mL; Sigma−Aldrich, Taufkirchen, Germany) and 10% fetal bovine serum (FBS; Sigma−Aldrich, Taufkirchen, Germany). The cells were maintained in standard conditions (a humidified atmosphere with 5% CO_2_ and 37 °C).

#### 2.9.2. MTT Assay

The antiproliferative activity of the OFS and OFG extracts was assessed using the MTT assay (Sigma−Aldrich, Budapest, Hungary). The experiments were carried out as described by Ghitu et al. [44]. The cells were seeded in 96-well culture plates at a density of 1 × 10^4^ cells/well and allowed to adhere overnight at 37 °C. The following day, the cells were stimulated with different concentrations of OFS and OFG extracts (10, 25, 50, 100, and 200 μg/mL) and incubated for 24, 48, and 72 h. The control is represented by the solvent dimethyl sulfoxide (DMSO), used for the stock solutions. After the incubation periods, 5 mg/mL MTT solution (Sigma−Aldrich) was added, and the plates were incubated for an additional 3 h. Lysis solution (100 μL) was used to dissolve the resulting formazan crystals, and then the absorbance was determined at 570 nm utilizing a microplate reader (Bio-Rad, xMark Microplate Spectrophotometer, Tokyo, Japan).

#### 2.9.3. Scratch Assay

The scratch test was performed to evaluate the antimigratory effect of OFS and OFG extracts on the invasion capacity of A375 melanoma cells [44]. The samples were also tested on HaCaT keratinocytes to establish the ability of the cells to migrate and to produce the repairing of the epidermal barrier affected by the injury. Briefly, cells were seeded onto 12-well culture plates at a density of 2 × 10^5^ cells/well until a confluence of 90% was reached. Then, a sterile pipette tip was used to draw scratches on the diameter of the well; the detached cells were washed with phosphate-buffered saline PBS (Thermo Fisher Scientific, Cambridge, MA, USA). The cells were stimulated with different concentrations of OFS and OFG extracts (10, 25, 50, 100, and 200 μg/mL). Pictures of the cells were captured at 0 h and 24 h using the Olympus IX73 inverted microscope provided with a DP74 camera (Olympus, Tokyo, Japan). Cell migration of the stimulated cells was compared to the control (no stimulation) and analyzed by the cellSense Dimension software.

The scratch closure rate was calculated using the following equation to quantify the migration ability of the cells [44]:

$$Scratch\;closure\;rate\;(\%)=\left[\frac{A_{t0}-A_{t}}{A_{t0}}\right]\times 100$$

*A_t_*_0_ is the scratch area at time 0, and *A_t_* is the scratch area at 24 h.

### 2.10. CAM Assay

The olive leavf extracts were subsequently submitted to an in vivo assessment to evaluate the tolerability upon topical application and to detect potential involvement in the angiogenesis process. The *in ovo* chorioallantoic membrane (CAM) assay was carried out [45]. As previously described by the authors [46,47,48], the fertilized chicken (*Gallus gallus domesticus*) eggs were incubated in a controlled humidified atmosphere at 37 °C. On the third day of incubation, a volume of egg white was extracted. Afterward, an opening was performed on the upper shells, resealed, and the incubation proceeded until the experimental procedure.

Investigation and analysis of the effects induced by the tested samples were carried out employing stereomicroscopy (ZEISS SteREO Discovery.V8, Göttingen, Germany). Image acquisition and processing were performed by Axiocam 105 color, AxioVision SE64. Rel. 4.9.1 Software, (ZEISS, Göttingen, Germany), ImageJ (ImageJ Version 1.54i, https://imagej.nih.gov/ij/index.html, accessed on 7 April 2024) and GIMP software (GIMP 2.10.36 revision 1, https://www.gimp.org/, accessed on 7 April 2024).

#### 2.10.1. Irritation Evaluation by HET-CAM Assay

The potential irritation induced by the olive leaf extracts’ administration upon chorioallantoic membrane tissues was assessed using the HET-CAM protocol [49], with minor variations being adapted to our laboratory facilities [46]. The assay allowed for semi-quantitatively evaluating the potential irritation of test compounds on the chicken embryonic egg’s chorioallantoic membrane by observing vascular events, including hyperemia, hemorrhage, and coagulation, immediately after the application.

To determine the irritative potential of the test samples, a volume of 300 µL of the olive leaf extract in a concentration of 100 µg/mL was pipetted on the vascularized membrane on day 10 of incubation and surveyed during a 5 min continuous stereomicroscopic observation of the modifications taking place on the vascular functionality of the CAM, mainly looking for abnormal events including hemorrhage, lysis, and coagulation. Following the 5 min monitorization of the treated CAM, the irritability score was calculated using the equation:

$$IS=5\times \left[\frac{301-Sec\;H}{300}\right]+7\times \left[\frac{301-Sec\;L}{300}\right]+9\times \left[\frac{301-Sec\;C}{300}\right],$$

where Sec H (hemorrhage) = first appearance (in seconds) of bleeding reactions on the membrane, Sec L (lysis) = first appearance (in seconds) of lysis of the vessel on the membrane, and Sec C (coagulation) = first appearance (in seconds) of the formation of coagulation on the membrane. 

We included in the study a positive control, sodium dodecyl sulfate (SDS); a negative control involved treatment with distilled water (H_2_O) and a solvent control, DMSO, in a concentration of 0.5%. All results were compared to the values indicated by Luepke [50], thus allowing us to identify the type of irritant for each test sample, as follows: 0–0.9—non-irritant, 1–4.9 weak irritant, 5–8.9 moderate irritant, and 9–21 strong irritant. 

#### 2.10.2. Angiogenesis Assessment Using the CAM Protocol

The potential implications of the tested extracts in the active process of angiogenesis were assessed on the developing vascular net of the chorioallantoic membrane of the chick embryo [45,51]. Extracts (100 μg/mL) were applied daily from day 7 of incubation in volumes of 5 μL inside plastic rings placed on top of highly vascularized areas of the CAM. The tested samples’ potential induced changes were investigated using stereomicroscopic live analysis and imaging. Selected images were further evaluated concerning the possible vascular architecture alterations and vessel branching enhancement or reduction. 

### 2.11. Statistical Analysis

The data obtained in the present study are expressed as mean ± standard deviation (SD). A one-way ANOVA test followed by a Dunnett’s multiple comparison test was used to compare groups. GraphPad Prism 10.2.2 (GraphPad Software, San Diego, CA, USA) was used for the statistical analysis (* *p* < 0.05; ** *p* < 0.01; *** *p* < 0.001; **** *p* < 0.0001).

## 3. Results

### 3.1. Identification and Quantification of Phenolic Compounds and Pentacyclic Triterpenes in OFS and OFG Extracts

Firstly, we intended to estimate the total amount of phytocompounds obtained by the hydro-ethanolic extraction. The yield of the dried extracts was therefore calculated, obtaining the following values: 9.46% for OFG and 11.34% for OFS. 

**Figure 1:**
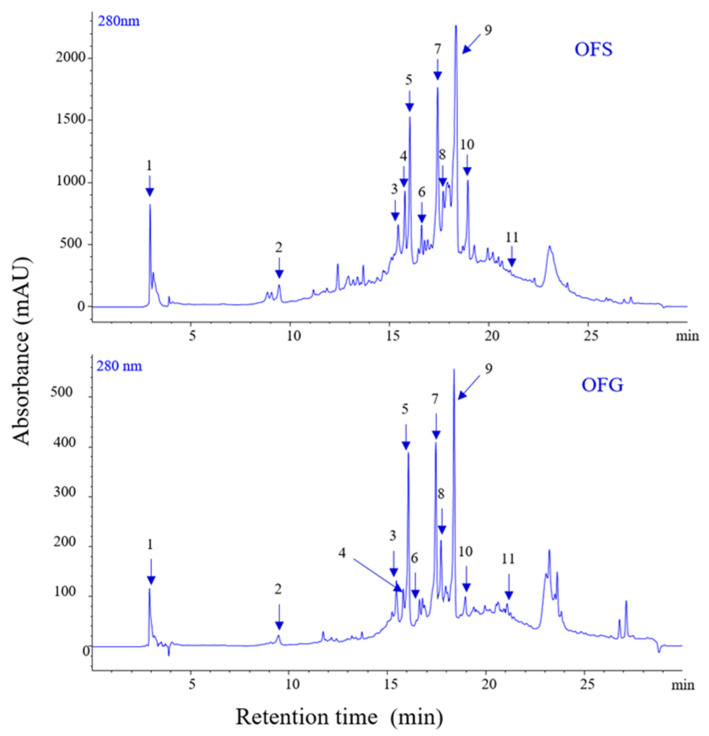
LC-PDA chromatograms of phenolics from OFS and OFG extracts recorded at 280 nm.

The extracts display different amounts of phenolic compounds, with OFG having a higher content than OFS. Figure 1 depicts the chromatograms of phenolics for the two extracts, and Table 2 presents the identification and quantification of the compounds. The total phenolic content for OFG is 99.228 µg/mg, whereas for OFS extract, it is 56.733 µg/mg, expressed as luteolin equivalents. 

**Figure 2:**
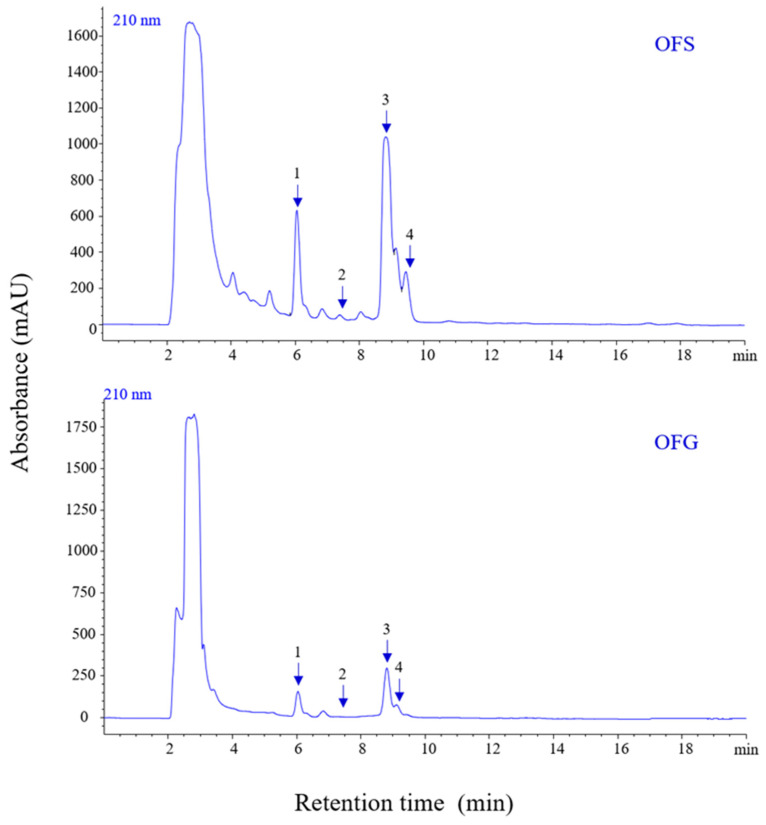
HPLC-PDA chromatograms of pentacyclic triterpenes from OFS and OFG extracts recorded at 210 nm.

Similar results were obtained after determining pentacyclic triterpenes in the extracts (Figure 2). In the case of OFG extract, the concentration of triterpenes was 111.747 µg/mg; for the OF from Spain, a smaller amount was obtained, namely 57.085 µg/mg (Table 3).

The phytochemical profile of the tested extracts highlighted the following aspects: luteolin 6-C-glucoside and luteolin 7-O-glucoside were the best-represented phenolic compounds in OFG (20.246 µg/mg and 20.959 µg/mg luteolin equivalent), followed by oleuropein (17.446 µg/mg luteolin equivalent) as the main components, whereas, in OFS, oleuropein was the main component (13.220 µg/mg luteolin equivalent). Furthermore, OFS contained important amounts of luteolin 7-O-glucoside (10.171 µg/mg luteolin equivalent), luteolin 6-C-glucoside (8.915 µg/mg luteolin equivalent), verbascoside (5.537 µg/mg luteolin equivalent), demethyloleuropein (5.069 µg/mg luteolin equivalent), oleuropein aglicone (4.890 µg/mg luteolin equivalent), luteolin-glucuronide (2.821 µg/mg luteolin equivalent), lingstroside (2.014 µg/mg luteolin equivalent), and tyrosol acetate (1.586 µg/mg luteolin equivalent). Likewise, OFG comprised similar phenolic compounds as the Spanish extract but in different quantities: verbascoside (8.877 µg/mg luteolin equivalent), luteolin-glucuronide (8.437 µg/mg luteolin equivalent), demethyloleuropein (5.271 µg/mg luteolin equivalent), oleuropein aglicone (5.257 µg/mg luteolin equivalent), lingstroside (4.354 µg/mg luteolin equivalent), luteolin (4.513 µg/mg luteolin equivalent), and tyrosol acetate (1.185 µg/mg luteolin equivalent). Regarding the total phenolic content, both extracts were characterized by a high amount of phenolic components (56.733 µg/mg luteolin equivalent for the Spanish extract, 99.228 µg/mg luteolin equivalent for the Greek extract, respectively). Complementarily, the main triterpenes found in the Greek and Spanish extracts were oleanolic (60.532 and 31.190 µg/mg extract, respectively), maslinic (33.813 and 15.873 µg/mg extract, respectively), and ursolic (16.093 and 9.066 µg/mg extract, respectively) acids. At the same time, betulin comprised a small amount of the total triterpenes content (1.309 and 0.956 µg/mg extract, respectively). Thus, OFG included a total triterpenic content of 111.747 µg/mg extract. In comparison, OFS was characterized by a reduced quantity of triterpenes (57.085 µg/mg extract) compared to the Greek sample.

### 3.2. Inorganic Elemental Content of OFS and OFG Extracts

The metal concentration determined in the two extracts varies, with the OFS having almost double the amount of elements compared to OFG. The metal content is presented in Table 4.

### 3.3. Characterization Techniques (TG-DSC and FT-IR) of OFS and OFG Extracts

**Figure 3:**
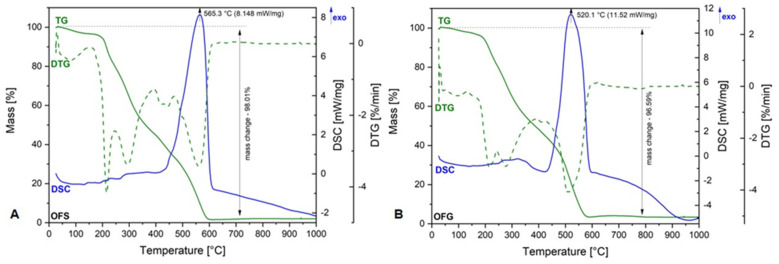
TG-DSC curves of OFS (**A**) and OFG (**B**).

Figure 3 depicts the TG-DSC curves of OFS and OFG extracts. 

As one can observe, both graphics are very similar, meaning there are no significant differences between the OF from Spain and Greece. Regarding the TG curve, the total weight loss percentage in both extracts was over 95%, which means that the entirely organic material is degraded at the end of the analysis. More exactly, the organic compounds’ degradation takes place at up to 600 °C. The total weight loss is accompanied by an exothermic process at 565.3 °C in the case of OFS and 520.1 °C in the case of OFG, respectively. These exothermic processes could be related to the degradation of aromatic compounds and carbohydrates in both OFs. The degradation process starts at around 200 °C in both cases, which means that, up to this temperature, we can say that the extracts are rather stable. 

**Figure 4:**
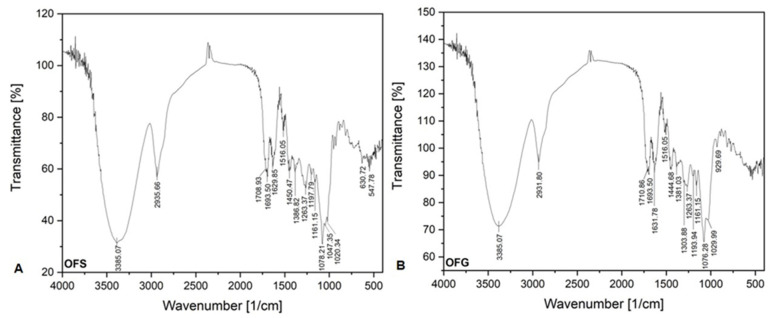
FT-IR spectra of OFS (**A**) and OFG (**B**).

Figure 4 shows the FT-IR absorption bands of both OFs. The FT-IR spectra of both extracts seem the same.

The relevant absorption peaks were recorded at 3385.07 cm^−1^ (strong broadband), corresponding to the O-H stretching vibration of hydroxyl groups from phenolic compounds (carboxylic acids). The bands around 2900 cm^−1^ correspond to the C-H stretching vibration, indicating the occurrence of the aromatic ring and alkyl group, respectively. The bands between 1690 and 1710 cm^−1^ correspond to the C=O vibration groups from carboxylic and conjugated acids. The medium weak multiple bands recorded between 1400 and 1650 cm^−1^ are attributed to the stretching vibration C=C groups from aromatic compounds. The bands around 1380 cm^−1^ correspond to the C-H bending vibration. It is very likely that the band at 1450.47 cm^−1^ from the OFS spectrum and the band at 1444.68 cm^−1^ can be assigned to the O-H bending vibration from carboxylic acids. The band recorded at 1303.88 cm^−1^ and the band from 1263.37 cm^−1^ could be assigned to the C-O stretching vibration from alkyl aryl ether groups. 

The bands between 1190 and 1047 cm^−1^, recorded at both FT-IR spectra, are attributed to the C-O stretching vibration. The band recorded at 1020.34 cm^−1^ from the OFS spectrum and the band recorded at 1029.99 cm^−1^ from the OFG spectrum are assigned to the =C-H bending vibration. The absorption bands under 1000 cm^−1^ are attributed to the out of plane bending vibration from aromatics alkenes =C-H.

### 3.4. The Antioxidant Activity of OFS and OFG Extracts Using DPPH Radical Scavenging Assay

**Figure 5:**
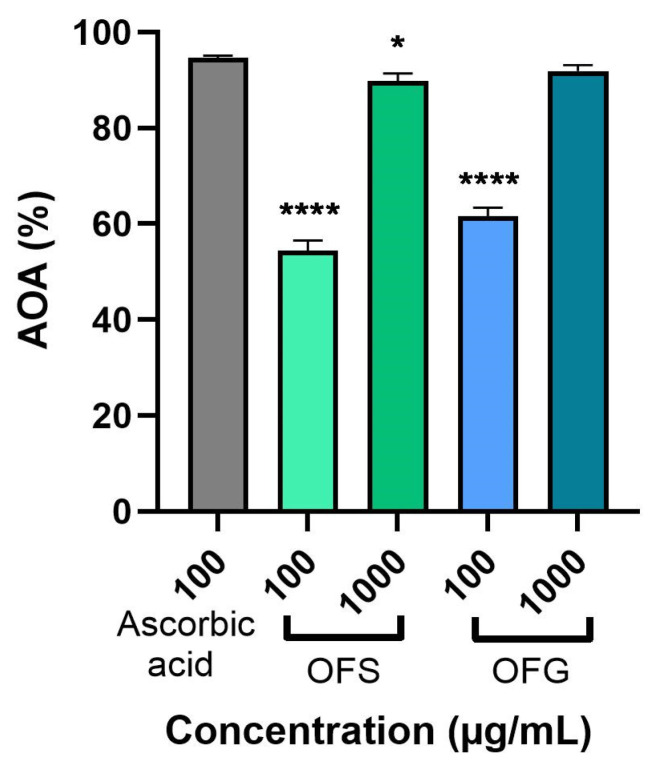
Antioxidant activity of OFS and OFG extracts. The data are expressed as mean ± SD. One-way ANOVA test followed by a Dunnett’s multiple comparison test was used to compare groups (* *p* < 0.05; **** *p* < 0.0001).

Figure 5 represents the extracts’ antioxidant activity (AOA) at 100 and 1000 µg/mL using the DPPH assay. The extracts were evaluated compared to the standard ascorbic acid (100 µg/mL). The data obtained indicate that both extracts elicited a significant AOA. OF from Greece had a slightly higher AOA compared to OFS (at 1000 µg/mL, the AOA for OFG was 91.89%, and for OFS, it was 89.97%), with both activities being close to that of ascorbic acid. 

### 3.5. EPR Measurements of OFS and OFG Extracts

**Figure 6:**
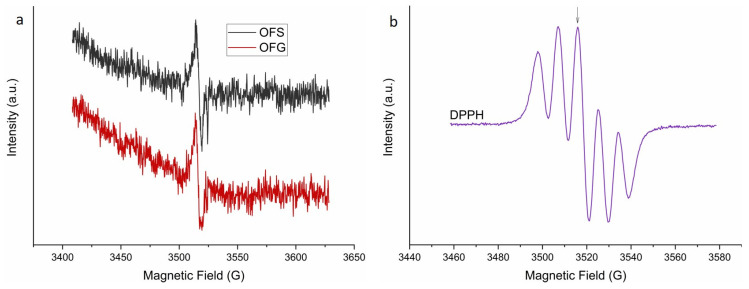
X-band EPR (9.882 GHz) spectra of the OF extracts with a 10 mg/L concentration (**a**) and characteristic 5-line DPPH (300 μM) EPR spectrum (**b**). The arrow indicates the position of the EPR peak at 3515.6 G, where the compound’s antioxidant activity was evaluated.

The EPR spectra of the OF with a 10 mg/L concentration are presented in Figure 6a. The EPR signal of the two compounds is identical, with a g-value of g_OLE_ = 2.0072, suggesting that the two extracts have similar radical compositions and concentrations.

**Figure 7:**
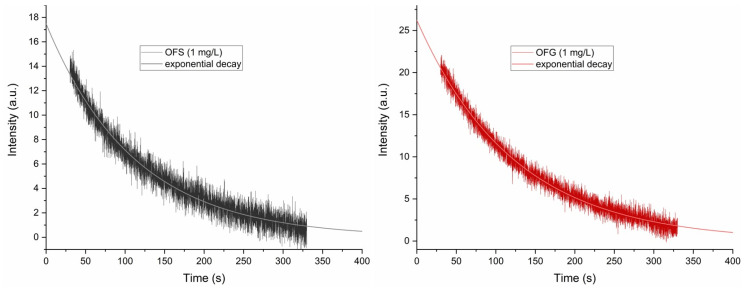
Exponential decay of the DPPH EPR-signal intensity at 3515.6 G in the presence of the OF extracts.

The antioxidant activity of the two extracts was evaluated using DPPH together with EPR spectroscopy. Figure 6b shows a characteristic 5-line DPPH EPR spectrum. The arrow indicates the position of the EPR peak, where the antioxidant activity of the extracts with a 1 mg/L concentration was evaluated, for which an intensity evolution of over 300 s was monitored after the extracts were added to the DPPH solution. The results of the antioxidant activity are presented in Figure 7. The measurements have a 30 s time offset, the time between when the OF extracts were added to the DPPH solution and the beginning of the EPR measurement.

Both extracts oxidize the DPPH so that, after 300 s, the DPPH EPR signal almost vanishes (see Figure 7). A monoexponential fit of the measured curves revealed the reaction constant of the antioxidant activity of the two compounds, with k_OFS_ = 8.95 × 10^−3^ s^−1^ and k_OFG_ = 8.1 × 10^−3^ s^−1^. The reaction constants also show great similarities, in agreement with the already described paramagnetic centers’ origin and concentration.

### 3.6. Antimicrobial Activity of OFS and OFG Extracts

#### 3.6.1. Disk Diffusion Method

The antimicrobial activity of the samples was evaluated on Gram-positive and Gram-negative bacterial strains and two *Candida* spp. (Table 5). The results obtained on the disk diffusion method are presented in Table 4. The minimum value for the antimicrobial activity of the tested samples was established at a diameter of 17 mm. Levofloxacin (LEV) and fluconazole (FCZ) disks were used for control.

The OFS extract elicited, at the highest dose tested (50 mg/mL), an antimicrobial effect against all microbial strains. The same extract displayed antibacterial activity at 25 mg/mL against the Gram-positive bacteria *S. pyogenes*, *S. aureus*, *E. faecalis*, and the fungal strains.

The OFG extract showed a similar or slightly lower effect at 50 mg/mL against the microbial strains compared to OFS and did not affect *P. aeruginosa*. At a lower dose, 25 mg/mL, OFG had an antibacterial effect only against *S. pyogenes*, *S. aureus*, and *E. faecalis*. 

#### 3.6.2. Determination of the Minimal Inhibitory Concentration (MIC), Minimal Bactericidal Concentration (MBC), and Minimal Fungal Concentration (MFC)

The broth dilution method evaluated the MIC and the MBC or MFC values (Table 6). The MIC value shows the lowest concentration, at which no visible growth of the strains takes place. MBC and MFC indicate the lowest concentration that reduces the amount of bacteria or fungi by at least three logs. 

The obtained data indicated that OFS and OFG elicited the best antibacterial activity against *S. pyogenes* (MIC = 12.5 mg/mL), followed by *S. aureus* and *E. faecalis* (MIC = 25 mg/mL). The results follow the disk diffusion method, with the lowest antibacterial activity obtained against the Gram-negative bacteria, *E. coli*, *S. enterica* serovar Typhimurium, and *P. aeruginosa* (MIC = 50 mg/mL).

### 3.7. The Antiproliferative/Cytotoxic Activity of OFS and OFG Extracts

#### 3.7.1. 3-(4,5-Dimethylthiazol-2-yl)-2,5-Diphenyltetrazolium Bromide (MTT) Assay

The effect of the OFS and OFG extracts was evaluated on a tumor cell line—A375 human melanoma cells and healthy cells—HaCaT human keratinocytes. The cells were incubated with different concentrations of the tested samples (10, 25, 50, 100, and 200 µg/mL) for 24, 48, and 72 h. 

**Figure 8:**
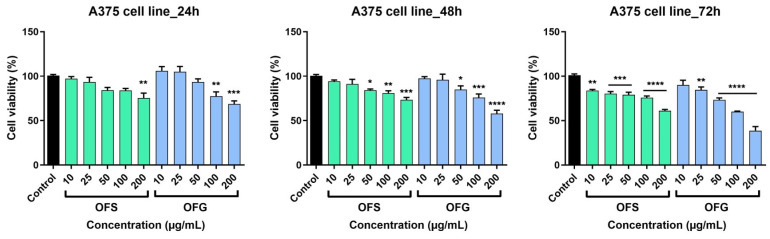
A375 melanoma cell viability after stimulation with OF extracts at different concentrations (10, 25, 50, 100, and 200 µg/mL). Results are expressed as mean ± SD. Comparison among groups was made using one-way ANOVA and Dunnett’s multiple comparison post-test (* *p* < 0.05; ** *p* < 0.01; *** *p* < 0.001; **** *p* < 0.0001 vs. control).

The extracts elicited a dose-dependent decrease in tumor cell viability, as shown in Figure 8. At 24 h, after incubation with the tested samples, OFG produced a slight increase in cell viability at the lowest tested doses, namely at 10 and 25 µg/mL (at 10 µg/mL, cell viability was 106.02 ± 8.2% and at 25 µg/mL cell viability was 105.06 ± 10.1% vs. control). The effect was not observed in the case of OFS extract at 24 h post-stimulation; at the lowest tested dose (10 µg/mL), cell viability was 94 ± 2.8%, and at the highest dose (200 µg/mL), cell viability was 73.2 ± 4.8% vs. control. The most significant anti-tumor activity was obtained following stimulation with OFG extract for 72 h (at 200 µg/mL, cell viability was 38.5 ± 7.9% vs. Control). For the OFS extract, at the same dose, at 72 h post-stimulation, cell viability was 60.8 ± 2.8% vs. control.

**Figure 9:**
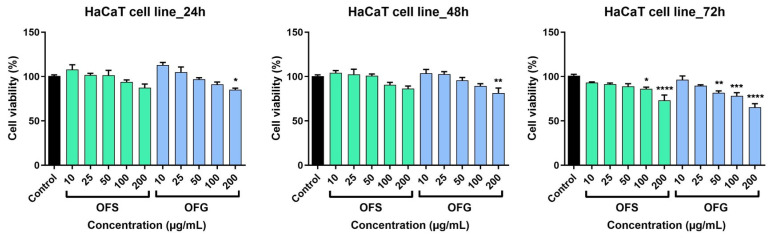
HaCaT keratinocytes viability after stimulation with OF extracts at different concentrations (10, 25, 50, 100, and 200 µg/mL). Results are expressed as mean ± SD. Comparison among groups was made using one-way ANOVA and Dunnett’s multiple comparison post-test (* *p* < 0.05; ** *p* < 0.01; *** *p* < 0.001; **** *p* < 0.0001 vs. control).

The human keratinocytes were less affected following treatment with the OF extracts (Figure 9). At 24 h post-stimulation, for OFS extract, a slight decrease in cell viability was recorded only at the highest tested doses; at 100 µg/mL, cell viability was 93.9 ± 3.8%, and at 200 µg/mL it was 87.4 ± 7% vs. control. OFG increased cell viability at 10 and 25 µg/mL; at 200 µg/mL, keratinocyte viability was 85.1 ± 3.1% vs. control. As in the case of the A375 cell line, the most significant effects were recorded at 72 h post-application of the OF extracts. A decrease in cell viability was observed; OFS provoked at the highest dose tested a viability of 73 ± 10.6%, whereas OFG at 200 µg/mL elicited a more potent effect, and the HaCaT viability was 65.2. ± 7.2% vs. control.

The OF extract reduced HaCaT cell viability, especially at the highest doses tested, but to a lesser extent than the effects obtained on the tumor cell line. These results indicate a slight selectivity of the OF extracts towards the cancer cell line. 

#### 3.7.2. Scratch Assay

**Figure 10:**
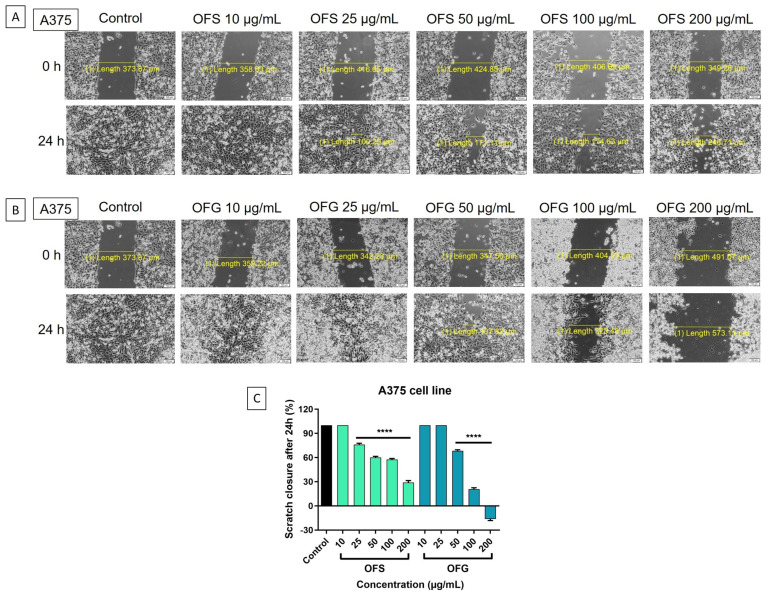
A375 human melanoma cells—microscopic images of the cells treated with the OF extracts (10, 25, 50, 100, and 200 μg/mL), initially at 0 h and 24 h; pictures were taken by light microscopy at 10× magnification. (**A**) Cells treated with OFS extract; (**B**) cells treated with OFG extract; (**C**) graphical representation of the scratch closure rate at 24 h post-treatment with OF extracts. Results are expressed as mean ± SD. Comparison among groups was made using one-way ANOVA and Dunnett’s multiple comparison post-test (**** *p* < 0.0001 vs. control).

A scratch assay was performed to evaluate the effect of the OF extract on cell migration. The control is represented by cells treated with a cell culture medium. In Figure 10, representative images of A375 cells are presented. A dose-dependent decrease in tumor cells migration was obtained after stimulation with the extracts. The most significant antimigratory potential was recorded for OFG extract at 200 µg/mL. 

**Figure 11:**
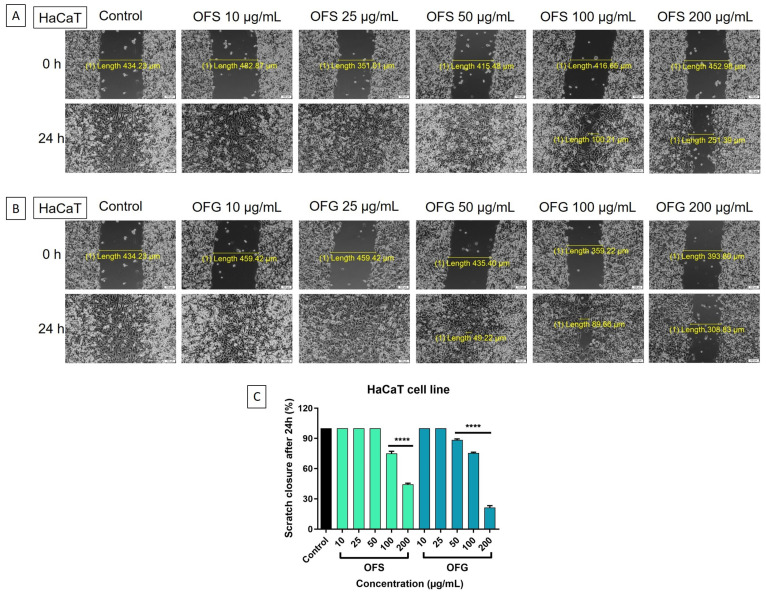
HaCaT keratinocytes—microscopic images of the cells treated with the OF extracts (10, 25, 50, 100, and 200 μg/mL), initially at 0 h and 24 h; pictures were taken by light microscopy at 10x magnification. (**A**) Cells treated with OFS extract; (**B**) cells treated with OFG extract; (**C**) graphical representation of the scratch closure rate at 24 h post-treatment with OF extracts. Results are expressed as mean ± SD. Comparison among groups was made using one-way ANOVA and Dunnett’s multiple comparison post-test (**** *p* < 0.0001 vs. control).

The effect on the HaCaT cell migration is represented in Figure 11. A similar pattern was observed in the case of the tumor cells, with OFG at the highest dose, 200 µg/mL, reducing cell migration. Nevertheless, the extracts influenced the HaCaT cells’ capacity to migrate to a lesser extent than the effect on the melanoma cells, thus indicating a selectivity in the OFS and OFG extracts.

### 3.8. The Anti-Irritant Effect of OFS and OFG Extracts Using the HET-CAM Assay 

**Figure 12:**
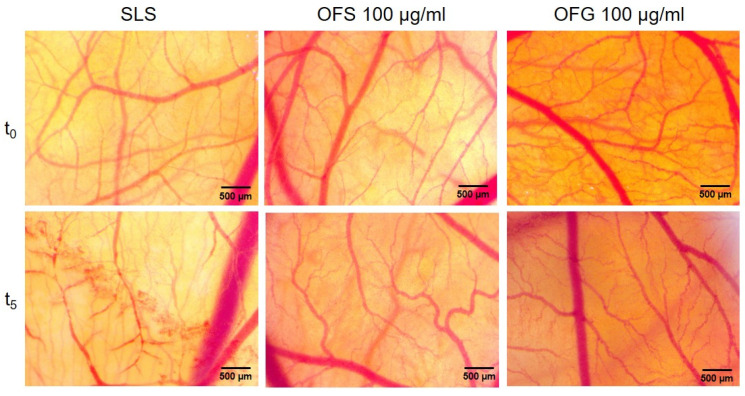
Stereomicroscopic images of OFS and OFG effects on the HET-CAM assay. The images were taken at the initial moment (t_0_) and 5 min (t_5_) after the application of the irritant solution (SLS 0.5%) or of the OF extracts (100 µg/mL).

Olive leaf extracts from Spain and Greece were evaluated in vivo, using the chorioallantoic membrane to assess the potential irritability towards mucosal or cutaneous tissues and check the biocompatibility of the two types of extracts with topical administration. The highest concentration of both extracts was applied in the HET-CAM assay. Compared to SLS, a strong irritant, no sign of toxicity in bleeding, coagulation, or vessel lysis was observed during the 5 min evaluation timeframe for the two olive leaf extracts in a concentration of 100 µg/mL (Figure 12, Table 7).

### 3.9. Modulation of Angiogenesis by OFS and OFG Extracts Using CAM Assay 

**Figure 13:**
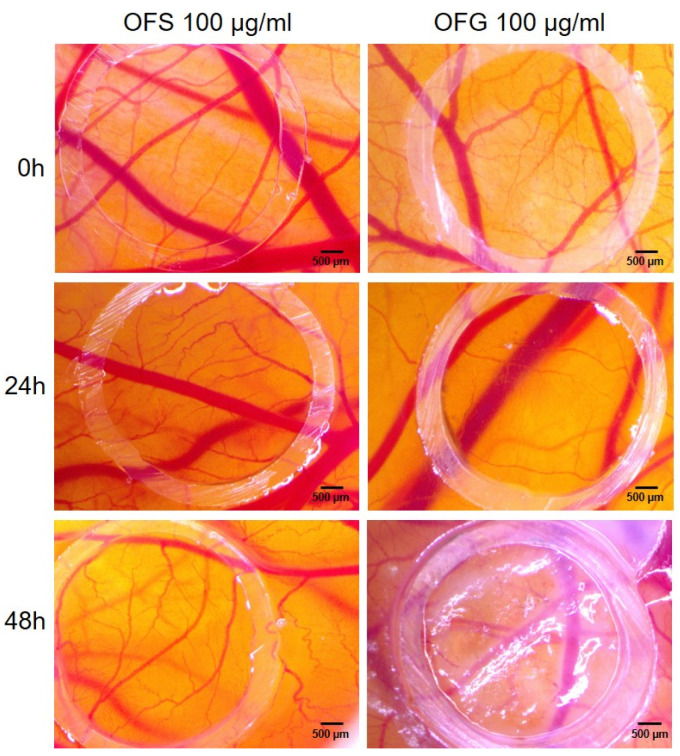
Stereomicroscopic images of OFS and OFG effects on CAM assay. Images were taken initially at 0, 24, and 48 h post-treatment.

The same extracts were submitted to investigate the potential effect on the active process of angiogenesis using the developing chorioallantoic membrane from EDD 7, undergoing an intense angiogenic process at this developmental stage. The extracts were tested at 100 µg/mL, previously shown as being as well tolerated for topical administration. As shown in Figure 13, the angiogenic process was affected, especially by the Greek extract, by reducing the number of new-forming capillaries on the application spot after 24 h post-administration. Conversely, the extract obtained from Spanish leaves did not affect the normal process of vessel formation at this stage of development.

## 4. Discussion

Olive is a tree that has multiple health benefits through the olive fruits from which the oil is extracted, but olive leaves have also received increased attention due to their composition, which is rich in antioxidant compounds, with multiple biological benefits.

As depicted in the Section 3, the olive leaf extracts were phytochemically characterized and showed important concentrations of phenolic compounds. The total polyphenolic content of the OFG extract (99.228 µg/mg luteolin equivalents) was almost twice that of the OFS extract (56.733 µg/mg luteloin equivalents). The highest content was found in the Greek extract for luteolin-7-O glucoside isomer with 20.246 µg/mg luteolin equivalents, followed by oleuropein with 17.446 µg/mg luteolin equivalents. The Spanish extract was dominated by oleuropein as main component but with a lower content compared to the Greek extract, of 13.220 µg/mg luteolin equivalents. Next to the polyphenols, triterpenic compounds were also quantified, and results showed that, for the Greek extract, total triterpenes (111.747 µg/mg extract) were almost twice as concentrated as in the Spanish extract (57.085 µg/mg extract). The main triterpenes in the OFG extract were oleanolic acid (60.532 µg/mg extract) and maslinic acid (33.813 µg/mg extract).

According to Silvan et al., OLE contained oleuropein (20,471 mg/100 g dry matter) as the main component, followed by verbascoside (6872 mg/100 g dry matter) and luteolin-7-glucoside (513 mg/100 g dry matter) [55]. Duque-Soto et al. analyzed the phenolic content of a commercial OLE and concluded that oleuropein represented the main phenolic component (76.1 mg/g dry extract) [56]. Benavente-García et al. also determined that the most abundant phenolic compound in olive leaf extract is oleuropein (24.54%—absolute content dry basis), followed by hydroxytyrosol (1.46%), luteolin-7-glucoside (1.38%), apigenin-7-glucoside (1.37%), and verbascoside (1.11%) [14]. Zaïri et al. established that the aqueous leaf extract of two varieties of *Olea europaea* L. obtained from Tunisia had a total phenolic content of 480.34 ± 1.36 mg GAE/g extract (Meski variety) and 546.06 ± 2.55 mg GAE/g extract (Chemlali variety), respectively. Both varieties contained oleuropein (74.512% and 85.672%) and verbascoside (6.881% and 6.035%) as main compounds, while the Meski variety was characterized by an impressive amount of luteolin-7-rutinoside (5.014%) compared to the Chemlali extract (0.902%) [57]. The hydroalcoholic extract of *O. europaea* var. *sativa* and var. *sylvestris* obtained from Tunisia showed values of total phenolic compounds of 5.04 ± 0.29 g GAE/100 g dry extract and 4.40 ± 0.38 g GAE/100 g dry extract [58], respectively. In contrast, OLE obtained from Brazil contained 131.7 ± 9.4 mg GAE/g dry weight of total phenols and 25.5 ± 5.2 mg/g dry weight of oleuropein [59]. Employing the HPLC-DAD method, Pereira et al. observed that Portuguese aqueous OLE contained seven phenolic substances, with oleuropein having the highest amount (26,471.4 ± 1760.2 mg/kg lyophilized extract) [60]. In the same vein, Hayes et al. determined that commercially available OLE purchased from Ireland encompassed oleuropein in the highest amount (1151.5 µg/mL), followed by verbascoside (68.6 µg/mL), luteolin-7-*O*-glucoside (25.6 µg/mL), apigenin-7-*O*-glucoside (15.9 µg/mL), tyrosol (15.6 µg/mL), and hydroxytyrosol (10.2 µg/mL) [61]. According to Muzzalupo et al., the HPLC characterization revealed that both Italian OLE (obtained using chloroform and ethyl acetate as solvents) contained, in descending order, oleuropein, luteolin-7-glucoside, luteolin-4-*O*-glucoside, and verbascoside [62]. Italian OLE obtained by De la Ossa et al. indicated a total phenolic content of 58.47 µg GAE/mg. The most abundant phenolic compound was oleuropein (32.64 ± 3.06 mg/g extract), followed by luteolin-7-*O*-glucoside (6.97 ± 0.24 mg/g extract), rutin (3.37 ± 0.33 mg/g extract), and apigenin-7-*O*-glucoside (1.97 ± 0.17 mg/g extract). At the same time, hydroxytyrosol, caffeic acid, and p-coumaric acid were present in lower amounts (<1 mg/g extract) [63].

Over the years, the scientific literature has acknowledged that oleanolic, ursolic, and maslinic acids represent the main triterpenes found in *Olea europaea* L. leaf extracts [21,64,65]. Thus, according to Somova et al., African OLE contained a 1:1 ratio of oleanolic and ursolic acids. In contrast, Greek and Paarl (Cape Town) OLEs were characterized by oleanolic acid in a percentage of 0.71% and 2.47%, respectively [66]. On the other hand, Duquesnoy et al. determined that triterpenic compounds represented 58% of unpurified hexane OLE (purchased from France), containing 12.5% oleanolic acid, 18.3% uvaol and 27.3% erythrodiol [67]. Taamalli et al. described the variation in total triterpenes according to harvesting time. The highest value of total triterpenes content was recorded in August and November (27.4 mg/g and 18.8 mg/g), while oleanolic and ursolic acids were found in the highest amounts regardless of the sampling time [68]. 

The antioxidant capacity was measured employing a DPPH radical scavenging assay. The obtained data showed that OFG possessed a higher radical scavenging activity than the Spanish one at both tested concentrations—100 µg/mL and 1000 µg/mL (OFG—61.06% and 91.89%, respectively; OFS—54.68% and 89.97%, respectively). The radical scavenging activity was close to that of ascorbic acid. The antioxidant capacity of the OLEs might be attributed to the synergy of the phenolic compounds and flavonoids, which are rich in electrons [14,69]. In the same line, methanolic OLE obtained by Ayoub et al. showed a significant radical scavenging activity of 89.44% at 1000 µg/mL [70]. Xie et al. also demonstrated that OLE obtained from China determined an increased antioxidant activity at 800 µg/mL (95.48%) compared to that of olive fruit extract (10.31%), mainly due to the redox potential of the phenolic compounds present in olive leaves [71]. Koca et al. showed that aqueous OLE possessed a stronger scavenging activity than the n-hexane extract at 1000 µg/mL (56.09% and 14.73%, respectively) [72]. Furthermore, 500 µg/mL aqueous extract of olive leaves provided from Tunisia exhibited a satisfying radical scavenging ability (~70%) [69]. Complementary, the DPPH assay showed that Meski and Chemlali varieties of *O. europaea* L. extracts exhibited increased antioxidant activity with IC_50_ values of 0.19 mg/mL and 0.13 mg/mL, respectively [57].

In addition to the beneficial effects of the classes of present polyphenols (flavonoids, phenolic acids, phenolic terpenes, tyrosols), the elements present in olive OFS and OFG extracts were studied. It is known that the content of elements varies depending on the species, climate, and area [73]. In the present case, two extracts from different areas with a Mediterranean climate were analyzed. Olive leaves are tools for monitoring atmospheric trace element deposition [74,75]. Hence, it is important to check the plant extracts regarding the metal content to ensure safety and being free of heavy metals. Olive oil production in Europe is conducted in Spain (63%), Italy (17%), Greece (14%), and Portugal (5%) [76]. Given the results for the two extracts evaluated in the present study, attention is drawn to the different compositions of elements. At first glance, we can say OFS concentrates almost double the amount of metals compared to OFG. In plant determination, elemental content has been considered individually, thinking only of the contribution of the respective element to the plant and the human organism and compared to recommended daily intakes and daily permissible limits [77,78,79]. The data obtained from these two extracts allow for the comparison of results and the searching for explanations of various repetitions. Thus, the obtained results are analyzed and correlated with the total phenolics, total pentacyclic triterpenes, and antioxidant activity, bearing in mind that these metals are complexed by plant phenolics. Accordingly, these metals are found in metal complexes called metallophenolomics. This is a pertinent observation according to the trend research in the field of metal complexes from plants, which have attracted attention recently [80].

In the present study, the GF-AAS technique detected the total metal content because the organic matrix was destroyed by acid digestion. If OFS is double the concentration in terms of metal content, it presents half the amount of phenolic compounds and triterpenes. An exception is tyrosol, which is higher in OFS than in OFG due to the low tyrosol chelation capacity [81]. This aspect can be understood since free polyphenolic compounds and free pentacyclic triterpenes were detected, not those chelated with metals. Where there are various metals, the free polyphenolic compounds chelate the metals, thus leaving less free polyphenolic compounds to be detected. This property of metal chelation by polyphenols also explains the olive’s resistance to the toxic stress induced by the polluting environment, as well as its adaptability in various acidic environments, where it is known that the concentration of elements is higher and more bioavailable for the plant. The support of this hypothesis is also proven by the DPPH method, which detects antioxidant activity, depending mostly on the number and position of hydroxyl moieties.

We would expect that OFS, which has half phenolic compounds, would have a lower antioxidant activity, but it shows a similar antioxidant activity that is detected both by the DPPH method and by the EPR Spectroscope. This similar antioxidant activity is also explained by the fact that the metal complexes of polyphenols also have antioxidant activity, even if they have fewer free -OH groups. For example, Xu et al. mention that quercetin complexed with metal ions shows excellent antioxidant activity, enabling flavonoids to oxidize by free radicals more easily than unmatched flavonoids. From a DPPH free radical scavenging test, they observed that quercetin combined with metals like calcium, magnesium, vanadium, copper, iron, cadmium, cobalt, calcium, and rare earth elements has a stronger scavenging capacity compared to pure quercetin, implying that the antioxidant activity of quercetin complexes is significantly higher than that of pure quercetin. Cu^2+^ complexes present higher activity on scavenging O^2−^ than Fe^2+^ or Fe^3+^ complexes. However, observing that metal complexes have enhanced antioxidant properties compared to the flavonoid ligand is not a rule [82]. Due to the abundant and complex composition of polyphenols and metal complexes, the identification of metal complexes from an extract is difficult to achieve due to the multiple possibilities of chelation of various metals with different ligands through simple coordination bond formation or redox reactions between the metal ions and the ligands. The attention of researchers is directed toward this because of the different properties and applications of flavonoid metal complexes. For example, improved complex photostability and increased fluorescence are obtained by coordinating Al^3+^ to 3-hydroxyflavone [83] or used as neuroprotective agents [84]. These aspects lead to a better understanding through further research.

Maximum allowed doses for herbal supplements are compared with the results to ensure the extract’s safety. Metal concentration varied from 0.0126 µg/g Cd in OFS to 5836.321 µg/g Al in OFS. Potentially toxic elements through plant exposure were represented by arsenic, lead, cadmium, nickel, zinc and copper. Pb, As, and Co concentrations were under the detection limit, more precisely under 7.4, 13.2, 5.4 µg/L, which meets the recommended criteria for a finished dietary supplement: maximum of 0.02 mg/day of Pb, 0.01 mg/day of As, 0.006 mg/day of Cd, and 0.02 mg/day of Cr [85]. According to the European Medicines Agency, the permitted concentrations of class 1 elemental impurities like Cd, Pb, and As are 0.5 µg/g (Cd) and 0.5 µg/g (Pb) [52]. Cadmium concentration in OFS was 0.0126 µg/g, while in OFG it was under the detection limit (0.1 µg/L); both of these are considered safe. Ni concentrations like 0.462 µg/g (OFG) and 1.766 µg/g (OFS) do not exceed the maximum oral concentration of 20 µg/g [52]. Zinc is a beneficial metal with antioxidant properties in the organisms, reaching the extract concentrations of 1.714 µg/g (OFG) and 6.500 µg/g (OFS). Compared to the recommended nutrient intake (RNI) 7200 µg/day, stipulated by the European Food Safety Authority [54], these do not have a valuable contribution to zinc uptake. Also, copper (2.257 µg/g (OFG) and 23.636 µg/g (OFS)) provides a low mineral intake, with precaution for the maximum oral concentration of 300 (µg/g) stipulated by the European Medicines Agency [52]. Chromium has good implications in diet regimens by playing a role in carbohydrate, lipid, and protein metabolism, enhancing insulin hormone, improving insulin sensitivity, and lowering glucose levels. An estimated safe and adequate daily dietary intake for chromium was set as an adequate intake (AI). The AI values for adults aged 19–50 are 35 µg/day (males) and 25 µg/day (females). OLE can contribute to chromium benefits with the following obtained concentrations: 7.833 µg/g (OFG) and 10.341 µg/g (OFS). Iron contents of 51.414 µg/g (OFG) and 69.398 µg/g (OFS) cannot bring value in terms of dietary intake, but, in regard to the complex form from the extract, OLE brings about strong beneficial effects on iron profiles in diabetes through mechanisms such as tempering oxidative stress that has resulted from an iron imbalance, chelating iron, and reduced protein glycation [86,87].

Al concentration raises some special attention because of the 3130.392 µg/g (OFG) and 5836.321 µg/g (OFS), with a 0.54 concentration ratio. For any concern, Al from extracts is safe due to the Provisional Tolerable Weekly Intake (PTWI) of 2 mg/kg bw/week, as people can consume up to this level without an appreciable risk to their health [88]. It is known that one of the richest plants in Al is *Camellia sinensis*, and it is also one with a high antioxidant capacity. Is this a coincidence? Tea plants with Al content as high as 17,000 mg/kg (dry weight) do not exhibit any toxic symptoms. Phenolic compounds were proposed as a potential ligand for chelating Al. Al detoxification and high Al tolerance may be because the phenolic-Al complex in plants is perhaps nontoxic. Most Al found in non-bioaccumulator species was present as a bound form rather than a free state, and about 60% of Al was localized in the apoplast, which comprises the intercellular space, the cell walls, and the xylem [89]. 

The mechanisms of Al accumulation in olive leaves are complex and require additional clarification. The compounds in OLE (polyphenols and metal complexes) may act synergistically with the body’s antioxidant system to defend against many diseases related to free radicals. 

Both tested OF extracts exerted important antimicrobial effects towards *Streptococcus pyogenes*, followed by *S. aureus* and *E. faecalis.* We noted a higher sensitivity of the Gram-positive strains to the tested olive leaf extracts compared to the Gram-negative ones. In agreement with the results obtained by others, a common mechanism could be observed with a different impact on the cell wall of the two types of bacterial strains [90]. Gram-negative bacteria are considered as more impermeable to antimicrobial agents and, thus, their resistance due to the absence of a lipopolysaccharide layer in their wall that is present in Gram-positive bacteria [91]. OFS acted as a stronger antimicrobial against all tested strains, while OFG had a lower effect and did not influence the growth of *P. aeruginosa* in a significant manner. Various research studies have been conducted to investigate the effectiveness of olive leaf extracts against certain microorganisms, specifically in terms of their antimicrobial and antifungal properties. Liu et al. observed an antimicrobial activity of hydroalcoholic OLE (purchased from Pittsburg, PA, USA) against *E. coli* (O157:H7 strain) and *S. enteritidis*. At 62.5 mg/mL, OLE completely inhibited the growth of *S. enteritidis*, while the *E. coli* strain was inhibited by 95% [92]. Sánchez-Gutiérrez et al. reported efficient antimicrobial activity of both aqueous and hydroalcoholic OLEs (obtained from Cordoba, Spain). The two tested extracts showed an inhibitory effect on *S. aureus* (CECT 5193), *S. typhimurium* (CECT 704), and *E. coli* (CECT 8295), with MIC values in a range of 2.5–40 mg/mL and MBC values in a range of 5–60 mg/mL, respectively [91]. Turhan et al. investigated the effect of different extraction solvents on the antimicrobial activity of olive leaves (collected from Mersin, Turkey). Researchers concluded that the aqueous extract delivered the best inhibitory effects against *S. aureus* (ATCC 25923), with an inhibition zone diameter of 29.4 mm [93]. 

Acetone OLE (olive leaves were collected from Mudanya, Turkey) was found to be very efficient against *S. aureus* (SA-11), *E. faecalis* (EF01), *S. typhimurium* (ST07), *S. enteritidis* (SE02), *E. coli* (EC-12), *P. aeruginosa* (PA-03), and *K. pneumoniae* (KP16), mainly due to the abundant concentration in oleuropein. MIC values obtained for the tested bacterial strains were 25–170 μg/mL, while MBC values were 50–340 μg/mL [94]. Aqueous Turkish OLE also exhibited antimicrobial effects against a series of pathogens such as *S. aureus* (ATCC 6538P), *E. coli* (ATCC 29998, ATCC 35218, O157:H7 RSSK 04054), *S. typhimurium* (CCM 3819), *P. aeruginosa* (ATCC 27853), *K. pneumoniae* (CCM 2318), and *C. albicans* (ATCC 10259). The obtained inhibition zones were situated within the range of 9–11 mm [95]. 

Furthermore, Zorić et al. demonstrated that oleuropein exerts antifungal activity against the fungal pathogen *C. albicans* (ATCC 10231). After conducting the antimicrobial susceptibility testing, the resulting MIC value was 12.5 mg/mL [96]. Polysaccharides extracted from Tunisian olive leaves displayed antibacterial activity against *E. coli* (ATCC 25922) and *Salmonella enterica* (ATCC 43972). The diameters of the inhibition zone around the wells were 10.5 ± 0.71 mm, respectively 23.5 ± 3.54 mm [97].

When tested on A375 human melanoma cells, the OF extracts had a dose-dependent decrease in tumor cell viability and proliferation; the Greek extract showed a more potent effect. The HaCaT human keratinocytes were less affected after applying the extracts than the tumor cells. Ruzzolini et al. observed that 200 µM (≈100 µg/mL) of enriched in oleuropein Italian OLE slightly reduced A375 melanoma cell proliferation during 72 h without affecting the viability of the human stem cells used as control. In comparison, higher doses (400 µM and 800 µM, respectively) significantly decreased the cell viability of both human cells. On the other hand, 200 µM extract exhibited an efficient anti-migratory activity on the A375 cell line, reducing the rate of closure of the wounds [98]. Moreover, the research team previously studied the cytotoxic potential of pure oleuropein using MTT and invasion assays. While 500 to 800 µM oleuropein reduced the A375 cell viability almost entirely, 250 µM (≈125 µg/mL) decreased cell viability up to 30%. The invasion assay showed that A375 cell motility was reduced following treatment with 250 µM oleuropein after 24 h of exposure compared to the untreated cells [99]. Mijatovic et al. demonstrated that a standardized dry OLE (acquired from Switzerland) possessed an impressive anti-melanoma potential against B16 murine melanoma cells and the A375 human melanoma cell line. The antiproliferative effect evolved in a concentration-dependent manner, reducing the cell viability to over 80% after 48 h of exposure [26]. Employing an MTT assay, Borugă et al. determined the cytotoxic effect of an ethanolic OLE provided from Albania containing luteolin and rutin as the main polyphenolic components. Hence, 100 µg/mL olive leaf extract reduced the A375 cell viability to 70% after 24 h [30]. Furthermore, De Cicco et al. published a recent study regarding the anti-melanoma effect of an ethanolic OLE (leaves were collected from Italy) against several melanoma cell lines, including the A375 human cell line and B16F10 murine cell line. The cytotoxic activity was assessed through MTT assay, while the anti-migratory and invasion potentials were evaluated using wound healing, invasion, and colony formation assays. The OLE exhibited a specific antiproliferative activity against melanoma cells, while the extract slightly affected normal human epidermal melanocytes (14.2%). Thus, melanoma cells’ viability was significantly reduced, with 63.3% for A375 and 74.5% for B16F10 cell lines at the highest dose (200 µg/mL). Additionally, 50 µg/mL extract markedly reduced the migration, invasion, and colony formation abilities of A375 melanoma cells over 24 h/48 h [100]. 

In order to be able to better characterize the anti-melanoma effect next to the potential selectivity on tumor cells, the Greek and Spanish olive leaf extracts were also tested on HaCaT cells, human keratinocytes. The results showed that HaCaT viability was decreased to a lesser extent compared to the effect obtained on tumor cells. Moreover, we showed through the microscopic images, which are in accordance with the viability assay, that the keratinocytes were less affected than the melanoma cells. Contributing to an extended anti-melanoma profile of the tested extracts, we evidenced minor inhibition upon healthy keratinocytes at the highest tested concentrations for which anti-melanoma effects were noted. Cádiz-Gurrea et al. investigated the effect of two Spanish olive leaves and fruit extracts rich in oleuropein against HaCaT cell line viability using an MTT assay. Whereas concentrations of 0.1, 1, 10, and 100 µg/mL of extracts did not produce significant changes in cell viability, the highest tested dose (1000 µg/mL) decreased HaCaT viability to 61.05% for 20% oleuropein extract and 42.06% for 30% oleuropein/10% triterpenes extract [101]. Machała et al. determined, employing a CCK-8 assay, that doses of 2.5–25 µg/mL OLE (purchased from Poland) showed no marked decrease in human dermal fibroblast cells (Hs68) [102]. Employing an MTS assay, Morandi et al. observed that doses of 300 µM of aqueous OLE (procured from Italy) moderately reduced HaCaT cell viability after 120 h exposure [103]. Similarly, 200 µM oleuropein showed a certain cytotoxic effect (26.2%) against HaCaT cells after long exposure (72 h) [104].

The tested olive leaf extracts were well tolerated on the vascularized chorioallantoic membrane and could represent safe ingredients for topical administration. Moreover, the extract obtained from Greek olive leaves reduced the developing vascularization of the CAM, with potential involvement in the modulation of a dysregulated angiogenesis process such as tumor angiogenesis. 

As identified by others, the active dose and composition of olive leaf extracts is an important feature of the biological effect on the cancer process and its microenvironment, indicating that different mechanisms are activated at different doses [25]. A modulating effect was observed by others, indicating that concentrations as low as 46.67 μg/mL induced a pro-angiogenic effect, while higher concentrations had an effect of reducing the angiogenic capacity [27]. 

In agreement with others [25,27], we found that the OFG extract at concentrations of 100 µg/mL showed a reduction in vessel branching at 48 h post-treatment. At the tested concentration, in correlation with a lower polyphenolic and triterpenes content and a less marked antioxidant effect, the OFS extract did not affect the normal vessel developing architecture. Our results, showing a reduction in the development of new vessel formation during a highly proliferative developmental stage of the embryonic capillary plexus, stand for beneficial use in limiting tumor invasiveness, as described by De Cicco et al., with OLE reducing melanoma spread by the suppression of epithelial to mesenchymal transition [100].

Furthermore, using the same in vivo experimental approach, we could evaluate the irritation potential of the tested extracts, given a possible future use in topical applications for dermatologic purposes. In correlation with the results for the in vitro assay on the HaCaT human keratinocytes, which showed an unimpaired migratory behaviour, both extracts were well tolerated in vivo, with good embryo viability and no irritation signs upon the chorioallantoic membrane.

Olive leaves from Spain and Greece were investigated as ethanolic extracts, OFG having higher concentrations of both polyphenols, such as oleuropein and lutein, and triterpenes, such as oleanolic acid and maslinic acid. Interestingly, inorganic elements were more concentrated in the OFS extract, indicating the potential chelating properties of highly concentrated polyphenols in OFG. OFS showed almost double the amounts of the inorganic tested elements and was especially high in Al and Fe. Furthermore, the extract with lesser inorganic content induced more pronounced effects in vitro in the form of antioxidants and in the form of selective cytotoxic agents on melanoma cells, also reducing cancer cells’ invasiveness. The potential antimigration effect of the OFG extract was also suggested by the antiangiogenic effect shown in vivo on the developing vessel plexus of the chorioallantoic membrane.

Moreover, our study contributes with data regarding the potential application of the OF extracts topically, in skin applications. The antimicrobial effects indicated good inhibition of *Streptococcus pyogenes* for both extracts. The two extracts were also positively evaluated concerning thermal stability. The safe use of the extracts was also confirmed in the HET-CAM assay, proving the absence of irritability when exposed to mucosal or epidermal tissues.

## 5. Conclusions

Overall, our findings contribute to a wider characterization of olive leaves originating from Spain and Greece, a valuable by-product in the olive harvesting process, with phytochemical and biological data indicating possible use as high antioxidant extracts with a potential impact on the healthcare system (through use as antimicrobial agents and as anticancer and anti-invasion treatment in melanoma). Both extracts have good phytochemical stability properties and are safe for topical applications, showing important antimicrobial effects towards *Streptococcus pyogenes*. When evaluating the biological effects, the high content of polyphenols and triterpenic acids of the Greek leaves could be correlated to the strong anti-radical capacity, a selective cytotoxic effect, as well as an antimigratory potential in regard to A375 melanoma cells and antiangiogenic effects on the CAM, thus recommending the profile of such an extract as a treatment to be further investigated in cancer studies.

## Figures and Tables

**Table 1:** Standard calibration curves for studied metals.

No	Metal	Wave, λ (nm)	Lower Limit (μg/L)	Upper Limit (μg/L)	Calibration Curve	R^2^
1	Pb	283.3	7.4	37	y = 0.001778 + 0.003524x	0.9994
2	As	193.7	13.2	58.1	y = 0.00185 + 0.001544x	0.9927
3	Co	240.7	5.4	29.4	y = 0.008353 + 0.010864x	0.9929
4	Cd	228.8	0.1	2.2	y = 0.004734 + 0.071971x	0.9923
5	Ni	232	4.2	34.6	y = 0.033774 + 0.011603x	0.9967
6	Mn	297.5	0.84	4,2	y = 0.007792 + 0.112496x	0.9925
7	Zn	213.9	1	8	y = 0.071658 + 0.092202x	0.9827
8	Cu	324.8	3.6	18	y = 0.020731 + 0.016628x	0.9961
9	Cr	357.9	5	22	y = 0.018371 + 0.018435x	0.9961
10	Fe	248.3	3.6	14.4	y = 0.02274 + 0.013974	0.9939
11	Al	309.3	13.2	58.2	y = 0.006978 + 0.00175x	0.9971

**Table 2:** Identification and quantification of phenolic compounds in OFS and OFG ethanolic extracts. R_t_–retention time; [M + H]^+^–molecular ion; UV λ_max_–maximum absorption wavelength in the visible region.

Subclass	Peak No.	R_t_ (min)	[M + H]^+^ (*m*/*z*)	UV λ_max_ (nm)	Compound	OFS	OFG
						Concentration (μg/mg)	
Tyrosol	1	3.05	181	274	Tyrosol acetate	1.586	3.185
Tyrosol	2	9.68	139	280	Tyrosol	1.132	0.682
Tyrosol	3	15.45	625	349.250	Verbascoside	5.537	8.877
Tyrosol	4	15.75	527	295	Demethyloleuropein	5.069	5.271
Flavone	5	16.08	449	350.249	Luteolin 6-C-glucoside	8.915	20.246
Tyrosol	6	16.64	525	320.240	Lingstroside	2.014	4.354
Flavone	7	17.54	449	350.249	Luteolin 7-O-glucoside	10.171	20.959
Flavone	8	17.82	463	350.250	Luteolin-glucuronide	2.821	8.437
Tyrosol	9	18.49	541	281	Oleuropein	13.220	17.446
Tyrosol	10	19.05	379	280	Oleuropein aglicone	4.890	5.257
Flavone	11	21.32	287	350.249	Luteolin	1.379	4.513
					Total	56.733	99.228

**Table 3:** Identification and quantification of pentacyclic triterpenes in OFS and OFG ethanolic extracts. R_t_–retention time; [M + H]^+^–molecular ion.

Peak No.	R_t_ (min)	[M + H]^+^ (*m*/*z*)	Compound	OFS	OFG
				Concentration (μg/mg)	
1	6.03	471	Maslinic acid	15.873	33.813
2	7.73	441	Betulin	0.956	1.309
3	8.81	455	Oleanolic acid	31.190	60.532
4	9.08	455	Ursolic acid	9.066	16.093
			Total	57.085	111.747

**Table 4:** Metal concentration (μg/g), as the mean of three determinations, elemental concentration ratio, and reference values.

Element/Sample	OFG		OFS		Element Concentration Ratio OFG/OFS	Reference
	Mean	SD	Mean	SD		
Cd	udl	na	0.0126	0.0015	na	0.5 [52] (a)
Ni	0.462	0.004	1.766	0.312	0.26	20 [52] (a)
Mn	0.933	0.044	1.478	0.072	0.63	11,000 [53] (b)
Zn	1.714	0.084	6.500	0.338	0.26	7200 [54] (c)
Cu	2.257	0.091	23.636	0.290	0.1	300 [52] (a)
Cr	7.833	0.146	10.341	1.018	0.76	1100 [52] (a)
Fe	51.414	0.307	69.398	5.003	0.74	45,000 [53] (b)
Al	3130.392	88.937	5836.321	233.660	0.54	2000 µg/kg bw (d)

udl = under detection limit; na = not applicable. Pb, As, and Co concentrations were under the detection limit. (a) Maximum Oral Concentration (MOC) (µg/g). (b) Recommended dietary allowance (RDA), µg/day/person. (c) RNI (recommended nutrient intake), µg /day, stipulated by the European Food Safety Authority. (d) Provisional Tolerable Weekly Intake (PTWI) of 2 mg/kg bw

**Table 5:** The inhibition diameters (mm) for the selected strains after incubation with OFS and OFG extracts (0.5, 1, 5, 25, 50 mg/mL).

	Disk Diffusion Inhibition Zones (mm)															
	*Streptococcus pyogenes*		*Staphylococcus aureus*		*Enterococcus faecalis*		*Escherichia coli*		*Salmonella enterica sv Typhimurium*		*Pseudononas aeruginosa*		*Candida albicans*		*Candida parapsilosis*	
Concentration (mg/mL)	OFS	OFG	OFS	OFG	OFS	OFG	OFS	OFG	OFS	OFG	OFS	OFG	OFS	OFG	OFS	OFG
50	27	25	24	22	22	20	20	20	20	19	19	17	22	21	21	21
25	24	22	21	19	19	18	12	10	10	10	10	9	19	17	18	16
5	15	14	12	10	10	9	10	8	7	7	7	7	10	8	10	9
1	10	8	9	7	8	7	8	8	7	7	7	7	8	8	7	7
0.5	7	7	7	7	7	7	7	7	7	7	7	7	7	7	7	7
LEV/FCZ	26	26	28	28	25	25	28	28	28	28	26	26	22	22	21	21

**Table 6:** The Minimal Inhibitory Concentration (MIC), Minimal Bactericidal Concentration (MBC), and Minimal Fungal Concentration (MFC) of OFS and OFG extracts.

Microbial Strains	OFS		OFG	
	MIC (mg/mL)	MBC/MFC (mg/mL)	MIC (mg/mL)	MBC/MFC (mg/mL)
*S. pyogenes*	12.5 12.5	12.5 12.5	12.5 12.5	12.5 12.5
*S. aureus*	25 25	25 25	25 25	25 25
*E. faecalis*	25 25	25 25	25 25	25 25
*E. coli*	50 50	50 50	50 50	50 50
*S. enterica serovar Typhimurium*	50 50	50 50	50 50	50 50
*P. aeruginosa*	50 50	50 50	50 50	50 50
*C. albicans*	25 25	25 25	25 25	25 25
*C. parapsilosis*	25 25	25 25	25 25	25 25

**Table 7:** The irritative scores of OFS and OFG extracts in the HET-CAM assay.

Test Compound and Controls	Irritation Score	Type of Effect
SLS 0.5%	16.43	strong irritant
OFS 100 µg/mL	0	non-irritant
OFG 100 µg/mL	0	non-irritant

Luepke scale: 0–0.9—non-irritant, 1–4.9 weak irritant, 5–8.9 moderate irritant, 9–21 strong irritant [50].

## Data Availability

The data supporting the findings of the study are available within the article.
